# Lived Experience of Treatment for Avoidant Personality Disorder: Searching for Courage to Be

**DOI:** 10.3389/fpsyg.2019.02879

**Published:** 2019-12-17

**Authors:** Kristine Dahl Sørensen, Theresa Wilberg, Eivind Berthelsen, Marit Råbu

**Affiliations:** ^1^Group Therapy Team, Aust-Agder County Outpatient Psychiatric Unit, Sorlandet Hospital, Arendal, Norway; ^2^Department for Research and Development, Clinic for Mental Health and Addiction, Oslo University Hospital, Oslo, Norway; ^3^Institute of Clinical Medicine, University of Oslo, Oslo, Norway; ^4^Aust-Agder County Outpatiet Psychiatric Unit, Sorlandet Hospital, Arendal, Norway; ^5^Department of Psychology, University of Oslo, Oslo, Norway

**Keywords:** avoidant personality disorder, treatment, psychotherapy, subjective experience, qualitative research

## Abstract

**Objective:** To inquire into the subjective experience of treatment by persons diagnosed with avoidant personality disorder.

**Methods:** Persons with avoidant personality disorder (*n* = 15) were interviewed twice, using semi-structured in-depth interviews, and the responses subject to interpretative-phenomenological analysis. Persons with first-hand experience of avoidant personality disorder were included in the research process.

**Results:** The super ordinate theme emerging from the interviews, “searching for courage to be” encompassed three main themes: “seeking trust, strength, and freedom,” “being managed,” and “discovering the possibility for change and development.” The main theme, “being managed,” included the subthemes: “getting a diagnosis,” “receiving medication,” and “attending therapy.”

**Conclusion:** Although this may not be specific to avoidant personality disorder, the findings highlight the importance of being met inter-subjectively as a person with intentionality and agency, even when one does not feel like one. The importance of establishing an emotional bond and emergent trust for open therapeutic collaboration, learning, and becoming able to build courage to begin to approach that which one fears is emphasized.

## Introduction

Avoidant personality disorder (AVPD) is characterized by fear of rejection and feelings of personal inadequacy, leading to extensive avoidance of social interaction, and is associated with significant distress, impairment, and disability (American Psychiatric Association, 2013; Lampe and Malhi, 2018). Despite AVPD being one of the most prevalent personality disorders encountered in clinical settings (Karterud et al., 2017), there is little research on specific treatment for this condition (Lampe and Malhi, 2018).

Various treatment and case studies of psychotherapy for AVPD indicate that psychological treatments may be helpful (Bartak et al., 2010; Weinbrecht et al., 2016; Lampe and Malhi, 2018; Simonsen et al., 2019). Examples of promising specialized therapy approaches for AVPD are cognitive behavioral therapy (Alden and Kazdin, 1989; Svartberg et al., 2004; Emmelkamp et al., 2006); metacognitive interpersonal therapy (Dimaggio et al., 2015, 2017; Gordon-King et al., 2018); emotion-focused therapy (Pos, 2014); acceptance and commitment therapy, combined with dialectical behavior therapy (Chan et al., 2015); interpersonal psychotherapy (Gilbert and Gordon, 2013); and short-term dynamic psychotherapy (Svartberg et al., 2004); as well as schema therapy (Bamelis et al., 2014). These specialized approaches spring from various theories of the core difficulties of AVPD and corresponding therapeutic strategies for adaptive change; however, it remains unclear if any particular forms of psychotherapy are more beneficial than others (Weinbrecht et al., 2016; Lampe and Malhi, 2018; Simonsen et al., 2019).

Although outcome studies have shown promising results regarding symptom reduction or other self-reported measures, it is unknown how these findings coincide with the clients’ personal views on their progress or on whether they felt supported in making positive changes (Katsakou and Pistrang, 2018). The concept of “symptom reduction” can in itself be seen as representing a medical view on personality disorders that has traditionally focused on finding “cures” for mental illness (Gillard et al., 2015). This medical view may lead us into a problem focused, and hence problem solving, view of psychotherapy, in which the therapist gathers information, forms ideas about solutions, and plans interventions to change, remediate, and heal the patient (Atwood, 1996). Furthermore, experiments, such as randomized controlled trials, can tell us something about whether treatment causes change but do not tell us why the variable or intervention led to change, as they do not address the question of which of the mechanisms and mediators the change occurred through (Kazdin, 2007).

The ongoing focus on how psychotherapy orientations, specific interventions, or therapist and client contributions influence treatment effectiveness has generated important knowledge but may direct our attention away from how clients experience therapeutic relationships and change processes (Levitt et al., 2016). Often the focus of psychotherapeutic research is on the delivery of techniques. Yet, techniques are necessarily used within the context of the relationship between the therapist and the client, which is influenced by the unique characteristics they both bring into the dynamic interaction, and which, over time, can facilitate new experiences and meanings (Clarkin, 2012; Shean, 2013). One way of approaching these questions is through qualitative research into the richness of the client experience of the process of therapy and treatment (e.g., Kazdin, 2007).

Persons diagnosed with AVPD, and personality disorders in general, have difficulties relating to others, as well as with their sense of self or identity. For example, individuals with AVPD have difficulties in identifying their own and other inner mental states, together with difficulty understanding that the states of others are not related to their own thinking (Moroni et al., 2016), a vulnerable sense of self and less self-reflexiveness, to help them regulate affect in relational contexts (Eikenaes et al., 2013). The use of avoidant dysfunctional defense responses may be related to efforts at preserving self-coherence (Bijttebier and Vertommen, 1999; Eikenaes et al., 2013) or to cope with fears of rejection (Lampe and Malhi, 2018).

One could question whether such difficulties warrant particular attention being given to the clients’ experiences of the relational context of therapy. To our knowledge, no qualitative study has been conducted specifically on the subjective experience of treatment for AVPD; however, there has been a substantial amount of research into the experiences of therapy by clients in general. In an extensive review of qualitative research studies into the clients’ experiences in therapy, Levitt et al. (2016), found that, at the core of all the themes investigated, regardless of therapy orientation, were clients’ experience of adopting an agential role toward holistic change within a therapeutic relationship of care and being known. Their experience of holistic change was related to curiosity about, and gradual self-attunement to, their own experience, and recognition of obstructive experiential patterns and unmet needs, as well as forming more adaptive alternatives. The therapeutic relationship would facilitate this exploration if it was flexibly structured and if clients experienced permission to be vulnerable and to discuss potentially threatening information, as well as to acknowledge difficulties and challenges to the relationship.

Qualitative studies of treatment and recovery, mainly for borderline personality disorder, reflect what these clients deemed most important for their development (Shepherd et al., 2016; Katsakou and Pistrang, 2018; Kverme et al., 2019); safety and containment, being cared for and respected, being an equal partner in treatment, and focusing on agency in practical and autonomous change, were all valued and important aspects of treatment. The authors understood these treatment characteristics as linked to the development of self-acceptance and self-confidence, through constructing new narratives related to their sense of self, as well as new ways of relating and feeling connected to others. Furthermore, these processes were seen as taking place within various social spaces, including professional relationships. These findings align with the recommendation by Levitt et al. (2016) to consider what the clients bring into therapy and to focus on how their experiences and potentials contribute to an interactive healing process.

Previously, we reported from our qualitative research project on the subjective lived experiences of AVPD as it relates to everyday life challenges and strategies (Sørensen et al., 2019). Their everyday lives came across as characterized by an ongoing struggle with sense making, sense of agency, and identity, as the participants both feared and longed for connection with others and described searching for a sense of self. Furthermore, this struggle seemed related to efforts at emerging as a relational person in a lifeworld of isolation that resulted in a sense of unresolvable intentions and left them bereft of options for resolving their relational challenges (Sørensen et al., 2019). Another research question within this project inquired into how persons diagnosed with AVPD made sense of their experiences with treatment and corresponding efforts at improving their condition. We understand “treatment” as all forms of formal therapy and treatment, ranging from specialized psychotherapy to more supportive therapy, medical treatment, physiotherapy, mindfulness and yoga approaches, skills training, and psychoeducational approaches, in all modalities that participants have taken part in.

As with other qualitative studies (Levitt and Piazza-Bonin, 2016, 2017; Levitt et al., 2017; Råbu and McLeod, 2018), it was necessary to publish separate papers on findings related to different research questions to present the rich qualitative data in sufficient detail.

The aim of the present study was to inquire into how persons diagnosed with AVPD made sense of their experiences of treatment.

## Materials and Methods

### Participants

#### Interviewees

The 15 interviewees consisted of nine women and six men who had received primary diagnoses of AVPD and were in treatment in outpatient hospital clinics in Norway. Their ages ranged from 20 to 51 years (mean = 33 years, SD = 9 years). Three participants lived with their children and a partner, three with a partner, two with their children, and seven participants lived alone. Four participants had completed their education at a primary level, nine at a secondary level, and two had finished a higher education. None of the participants worked at the time of the interviews and all received welfare. Two participants were taking part in their first course of treatment, two had undergone 2–3 courses of treatment, and 11 participants had taken part in three or more courses of treatment. Their treatments varied from individual therapy to specialized group therapies and psychomotor physiotherapy in outpatient settings and individual therapy in private practices. Therapists were psychiatrists, clinical psychologists, or psychiatric nurses. In addition, several participants had sought help *via* religious guidance, yoga, or meditation practices.

#### Researchers

KS is a clinical psychologist and PhD student. TW is a psychiatrist and professor. EB is a theologian with a PhD in Health Sciences, who mainly works as a hospital priest. MR is a clinical psychologist and associate professor. Together, the clinicians had backgrounds in schema therapy, mentalization-based therapy, relational and dynamic therapy, and psychotherapy integration. All researchers share an interest in qualitative research into the subjective experience of various phenomena of everyday life and more specifically of personality disorders and therapeutic processes.

#### Service-User Involvement

We included persons with first-hand experience of AVPD throughout the research process, to increase the quality, relevance, and ecological validity of the study (Borg and Kristiansen, 2009; Veseth et al., 2013). A coresearcher collaborated with the first author through all stages of the research process. We also established a reference group consisting of two service users, two experienced clinical psychologists, the first author, and the coresearcher who met regularly to discuss and reflect upon the research questions, the interview guide, and the emerging themes.

### Procedures

#### Recruitment

We purposively recruited participants who had received a primary diagnosis of AVPD by their respective therapists, using the Structured Clinical Interview for DSM-IV Axis II Personality Disorders (SCID-II; First et al., 1997). Second, we purposively strived to recruit men and women of various ages and with various treatment experiences, regarding length, modality, and type (specialized for personality disorder or regular outpatient treatment offered at site), provide variable subjective experiences, and reflect common clinical reality. All participants were recruited from the same hospital but at various outpatient sites within the same region.

Nine participants from the research project, “An Examination of the DSM-5 Level of Personality Functioning Scale in a Representative Clinical Sample” (Buer Christensen et al., 2018, 2019) who had agreed to be contacted at a later stage for additional research purposes were approached by the first author (KS). They received written information about our research project through their therapists. Five persons agreed to participate and were interviewed.

Ten participants were recruited and interviewed through reaching out to therapists at various outpatient clinics. The therapists were given written and oral information about the research project as well as brochures to give to patients with a primary diagnosis of AVPD. Those who expressed interest in participating were contacted by the first author by phone or text message and received written and oral information about the project.

#### Interviews

The first author, the coresearcher, and the reference group developed a lightly structured and open-ended interview guide to ensure that the subjective experience of the participants could be represented faithfully. We conducted a pilot interview to receive feedback from a participant on how the interview situation was perceived and then slightly revised the interview guide according to the feedback. The first author conducted two in-depth, face-to-face interviews, lasting 60–90 min each. The interview guide was primarily used in the first of the two in-depth interviews. We included a second interview to improve rapport and give the opportunity to elaborate on topics introduced in the first meeting. The second interview took place approximately 2–3 weeks after the first.

Questions that related to experiences with treatment and efforts to improve their condition were What have you done to get better? Can you tell me about the treatments you have taken part in so far? What has been useful/less useful in your treatments so far? What do you think you need to get better?

A preliminary analysis of the first interview by the first author and the coresearcher formed the basis of the follow-up questions for the second interview. For nine of the participants, the coresearcher read the anonymized transcripts and gave feedback to the first author on her reflections and suggestions for further questions regarding important areas in the second interview.

Although most participants conveyed their inexperience in articulating and reflecting in an interpersonal setting, such as in-depth interviews, the questions related to treatment experiences seemed of importance and accessible to them, and they appeared to be open to sharing their views on their treatments; however, the question “What do you think you need to get better?” elicited few responses. A rephrasing of the question to “If you got better, how would you be and what would you do?” resulted in fuller descriptions. Perhaps a shift in focus from a mode of psychological problems to a possible future in which the participants imagined feeling content gave access to increased attention to their wishes, likes, and dislikes. When participants referred to theoretical concepts, the interviewer would prompt them to try to explain their views in their own words what they understood those concepts to mean.

We made efforts to ensure the wishes of participants for privacy and comfort, and the interviews took place at their site of choice. Thus, 11 participants were interviewed in an office at their hospital unit, and four were interviewed in their homes.

The interviews were audio recorded and transcribed verbatim for analysis. All transcripts were verified once and imported into NVivo software (QSR International, 2015) for principal analysis.

### Qualitative Methods

#### Data Analysis

As we position ourselves within a research tradition that views meaning as “something that exists within human subjectivity, rather than on the plane of material nature” (Atwood and Stolorow, 2014, pp. 3–4), our analysis found its base in phenomenological hermeneutical epistemology, through being based in Interpretative Phenomenological Analysis (IPA; Smith et al., 2009). Within this approach, we understand subjects as embodied beings embedded in a social and cultural world (Zahavi and Martiny, 2019). IPA is phenomenological through its concern with the perception of objects or events by individuals and hermeneutic through its recognition of how observations are always interpretative (Smith et al., 2009; Larkin and Thomson, 2011).

The analytical steps of IPA recognize this dynamic movement of descriptively giving voice to the concerns of the participants represented in the transcripts and the interpretation involved in contextualizing and making sense of these concerns from a psychological perspective (Larkin et al., 2006). Hence, the first analytical phase involves trying to understand the participant worlds through focusing on the subjective experiences conveyed in the transcripts. This phase is commonly characterized by efforts at capturing the essential elements of the transcripts (Smith, 2015). Subsequent analytic phases are increasingly interpretative and often informed by existing theoretical constructs, trying to offer interpretative accounts of what it *means* for participants to have their concerns, within their particular contexts (Larkin et al., 2006, p. 113). In doing so, the researcher may move from descriptive to increasingly higher levels of abstraction, which allowing production of a theoretical framework. The analytic account is thus based on, but may transcend, the participants’ own terminology and conceptualizations, providing it can be traced back to a core account and related to a particular research question (Smith, 2004; Larkin et al., 2006). To remain aware of the movement between descriptions and interpretations, we emphasized explorative and reflexive aspects of hermeneutic phenomenology throughout the research process (Finlay, 2008; Binder et al., 2012).

We read and reread each transcribed interview to become familiar with the data. Next, text-segments related to the research question of the subjective experience of treatment for AVPD were separated into corresponding broad content units for each participant. These content units were coded on content meaning. Next, we suggested abstractions of emerging themes for each case before making comparisons in a cross-case analysis, generating suggestive themes at a group level. This stage involved highlighting similarities and variance while considering recurrence across individual interviews. Thus, we deepened our understanding of segments that appeared to consist of more superficial descriptions yet became enriched when considered in light of segments drawn from the interviews of more articulate participants (Kirkevold and Bergland, 2007). Finally, the emergent themes were organized into a superordinate theme that captured the inferred overarching meaning, which organized the final main themes and subthemes, resulting in consensus regarding the presented version.

#### Credibility Checks

We integrated several credibility checks during the analysis to assess the accuracy of the findings. We asked each participant to add any information that might have been omitted or if there was any information that we should have asked about. We ensured reduction of researcher bias and maintained an active user perspective through meetings and reflections of the coresearcher and the resource group. Research analysis was continuously reviewed by the researchers, KS and MR, to ensure that multiple perspectives were included in the understanding of the complex nature of the data while still reaching a consensus regarding the interpretation and resulting themes. KS conducted validity checks of the themes by returning to the original transcripts repeatedly during the analytical process, ensuring that the interpretations and empirical data were consistent. Finally, KS contacted the participants and asked for feedback on a draft of this article and a summary of the findings. Seven participants responded to our request. The participants expressed how the themes resonated well with, and captured, their experiences. Some said that it was good to see that they were not alone in their experiences and that to be understood in treatment was of particular importance. In addition to remarking that the findings reflected similarities among them, they also noted that different persons had varying experiences of treatment; for example, not everyone had attended group therapy.

#### Reflexivity

As we base ourselves within a phenomenological-interpretative understanding of human experience, we also see the researcher as “unavoidably present and influential in the inquiry” (Gemignani, 2017, p. 185). The role of the researcher as an active participant in the construction of knowledge warrants reflexive attention to the researchers’ positions and perspectives, as well as to personal responses and biases throughout the research process (Finlay, 2002).

When analyzing text segments related to subjective experience of treatment and efforts to change, the emerging themes seemed in particular to draw our attention to the concepts, “therapeutic change” and “treatment.” How have we come to understand therapeutic change and treatment? We often use the words treatment and measure outcome in ways that do not consider clients’ subjective meaning of change. Rather we predefine and operationalize change into outcome measures that assume alignment between the experiences of the observer and the observed’s experience of being helped. This view may represent the influence of the ongoing discourse and performances of current psychotherapy practices (Gemignani, 2017). The researchers hence strived to stay aware of the assumption that the participants wanted to change themselves in theoretically predefined ways and thus to remain open to the participants’ experiences.

### Ethical Considerations

Due to the potential vulnerability of the participants when talking about sensitive topics in the interviews, we took efforts to secure comfort and support for the participants. The interviews took place at site of their choice and were conducted by the researcher, KS, who is an experienced clinical psychologist and psychotherapist. In addition, participants were all in therapy at the time of the interviews to ensure the availability of prolonged support in case of need. All participants gave their signed informed consent to participate. The project was approved by the Regional Committees for Medical and Health Research Ethics (REK Sør-Øst 2015/980).

Biographical details were changed slightly to ensure anonymity.

## Findings

All participants described having sought help in life phases of overwhelming frustration over their anxieties, worries, and on-going ambivalence of longing for and fearing connection to others and themselves, as their coping strategies had not brought sufficient relief to allow them to continue enduring their situations.

Our analysis supported an overarching superordinate theme, “searching for courage to be.” This superordinate theme represents the most abstract level of analysis and incorporates the main themes, “seeking trust, strength, and freedom,” “being handled,” and “discovering the possibility for change.” These main themes pertain to all participants but also include important variance and nuances of the participants’ experiences of treatment.

The representativeness of our findings and the recurrence of themes across individual cases are indicated by the frequency labels *general*, *typical*, and *variant*, as suggested by Hill et al. (2005). The main themes were all *general*, in the sense that they applied to all cases, or all but one case, and are referred to in the text as *all participants.* The themes considered *typical* applied to more than half of the cases are referred to as *most participants.* Variance within themes was represented by less than half, but more than two, cases. This is reported as *some participants* in the text.

Themes are illustrated below by quotes from individual participants.

### Searching for Courage to Be

Our analysis led to the superordinate theme, “searching for courage to be,” which encompassed the various experiences of treatment described by the participants. The title of the superordinate theme was inspired by the book title, “The courage to be,” by Paul Tillich (1886–1965), but without further reference to his philosophy or theology. That is, beyond the beautiful quote: “The courage to be is the courage to accept oneself as accepted in spite of being unacceptable” (Tillich, 1952, p. 164), and a resonating sense of how daunting a search for such an acceptance can be, which seemed to permeate the themes on all levels.

The participants had sought help that they hoped would support their search for courage to start resolving their insecurities and fears, wanting to become able to understand and relate to themselves and others, as well as to better manage their everyday lives. Most participants still searched for the help they needed, telling stories of how they did not yet feel fully understood, or found a way to make sense of, or begin to resolve, their struggles; however, all participants also told of stories or incidents of trust and understanding that seemed connected to their own sense of possible resolution and development. Those who had more experience with trust and understanding in their therapeutic relationships told of toil and movement toward emerging integrity in their way of being (Table 1).

**Table 1:** Overview of the main themes and subthemes.

•Searching for the courage to be
•Seeking trust, strength, and freedom
•Being managed: getting a diagnosis, receiving medication, and attending therapy
•Discovering the possibility for change and development

*The main themes are general and pertain to all participants*.

#### Seeking Trust, Strength, and Freedom

All participants described that their goals were to find greater self-confidence and inner strength and to become able to stand their ground and cope with adversities, without feeling as if they would fall apart.

“I need inner strength and to become able to trust and believe in myself. I have to trust that I am as valuable as everyone else, kind of equal. I just wish to be well.”

They also described wishing to know their likes and dislikes and live more by them, feeling free to do what they wanted without being afraid of others’ possible evaluations and reactions. At the same time, they wished to feel included in the world, to feel joy and happiness, or just be ok.

The participants did not long to get many friends but wished to have some good ones to talk to and to do things with. They wished to be able to support themselves and work, to travel, or to just go shopping, or to places, they had heard of. To become able to reach their goals, they wanted to get to know themselves and know what to do to improve their condition; however, to approach such goals and wishes implied facing several fears: the risk of potential rejection, the risk of not being taken seriously, and the risk of someone not believing you. They would also need to take in the full scope of their condition, of the possibility of failing or being exposed, and of all the insecurity that follows from entering the unknown. Subsequently, some participants said that asking for help implied that you had to acknowledge that you had mental problems, something that seemed associated with both shame and defeat.

“I would rather manage on my own so I say that I am fine. I may have difficult days at home, but then when I get to the clinic, I say that I am ok. I do not want to be that kind of person that does not dare to do things.”

This was described as a barrier for them. They thus hoped to improve by themselves, stalled the initiation of therapy, or downplayed the seriousness of their condition when being assessed by health professionals. When impelled to overcome these barriers, there were various paths into treatment. Some participants had initiated contact with their general practitioners themselves who then referred them to further treatment. Others had been advised or pressured to seek help by family or friends who had become aware of their struggles and worried about them. Most had received various forms of help and treatment on their path to relieve their struggles, ranging from religious support, to prescription drugs, physiotherapy, meditation, and to more or less specialized psychotherapy. Despite the barriers and various ways into and through treatment, all participants described seeking help to resolve questions of how to be and what to do, which could alleviate their struggles.

#### Being Managed

The experience of treatment that came across as most salient for all participants was a conveyed sense of being managed or handled within the treatment contexts in which they participated. This sense of being managed was not expressed as something that they necessarily considered negative or felt opposed to. Rather, they seemed to have entered treatment with a hope of receiving explanations and directions given by a professional that could understand and somehow prescribe relief; however, a sense of discontentment evolved, becoming more noticeable when participants progressed into what seemed to be a more established relational pattern of staying detached within their treatment setting. Their disconnection seemed related to descriptions of interplay between not feeling able to make themselves understood and not feeling understood, in a way that maintained an experience of simultaneously being inactive and being told what to do. Therefore, although being managed often was initially what they wished for, they described becoming discontent and left wanting, as the treatment progressed. This pattern seemed to emerge within the context of getting a diagnosis, receiving medication, and attending therapy.

##### Getting a Diagnosis

All participants said that they had received several diagnoses; most were first diagnosed with various anxieties and depression, and subsequently with AVPD. The experience of being diagnosed was, for most, described as one of finally being understood, and as giving hope in that their challenges were recognized as something that could be explained and treated. Although the diagnosis of AVPD brought relief, most participants expressed concerns about how to make sense of this diagnosis, in terms of what to do or how to be. Unresolved issues of how to deal with a diagnosis of one’s personality seemed to span from not wanting to be the kind of person that the diagnosis described and fears of having to conform to all characteristics of the diagnosis, to bewilderment over what they should do when diagnosed.

“This diagnosis, there is a lot that falls into place about how I have related to things. But I think it is pretty normal to have those traits and it is this ambivalence towards this being a problem and me being sick needing treatment or this just being the way that I am, it’s my personality and I just have to accept it.”

Most considered the diagnosis of AVPD more like an explanation of why they had felt so depressed and anxious, rather than an acceptance of *being* disordered. Some described that they felt that they were not able to develop or express the person they truly were or could be, due to their fears and insecurities and that the diagnosis did not reflect their true self.

##### Receiving Medication

All participants described how they had considered medication, and most had tried prescription drugs, mainly antidepressants. Most participants conveyed how they did not ascribe improvement through medication as something initiated from within or assigned to their own sense of mastery. Rather they considered medication to be an effective way to create distance from their painful thoughts and feelings, making them easier to suppress and thus leaving them able to work or move through their everyday lives.

Some participants described how their medication had actually made them fare worse than before, leading to states of apathy, loss of vitality, desire, and creativity. Others described their medication as necessary, as they pictured themselves not managing their everyday life or just falling apart without it.

After some time, the participants who had decided to use medication described how they became uncomfortable with this disconnection from what they knew their inner states to truly be, feeling like they moved away from who they were, loosing themselves even more than before.

“Medication (antidepressants) makes it somewhat artificial. It is just like putting your problems inside a locker and throwing away the key without doing anything about them. It kind of works and makes you function. Then, it did not work, and I just quit because I thought it could not get any worse.”

They thus conveyed that, even if their functioning improved, they did not feel like they actually fared any better and still felt unhappy about their condition.

##### Attending Therapy

All participants described aspects of their former therapies that conveyed a sense of becoming passive receivers of others’ evaluation and being taken care of at others’ will. This managing left them inactive, discontent, and detached, like a victim of their circumstances. They did not state that their therapists had bad intentions or were there to mistreat them. Rather, there was reduced connection, or an absence of common sense making, that could have resonated with their own experiences.

“My general practitioner sent me here and I did not know why. I thought this was a place for crazy people. The therapist I got did not understand me or what I said. It was kind of tiresome. I sat there, nodded and smiled and she talked and then I talked a bit and that was it. I had no idea what she talked about most of the time. I just kept my thoughts inside.”

Some participants described how the therapist had regularly asked questions like, “How would you describe your problems?” or “What are your goals?,” which they did not know how to answer. Mere questions about their thoughts, feelings, or opinions about something could leave them quiet. Either they did not know the answer, felt unsure about the right thing to say, or they found it frightening or uncomfortable to talk about themselves.

“My problem is that I always am like: OK, if I am to sit and talk to someone, then what should I say? What should I talk about and why?”

Some therapists were described as being too “professional,” meaning that the therapist was conceived as unwilling to engage in conversation that was more personal or to express their own thoughts and opinions. The therapists were experienced as parrying the participants’ efforts at being polite or gaining advice, by asking for the participants’ own thoughts or opinions, or by presenting interpretations of the participants’ conversational efforts that seemed out of place.

Some participants described their former therapies as mostly consisting of talking about what had taken place since their last session. Some said that they had found it so uncomfortable to talk about themselves that this talking did help them to get a bit more used to it. They described talking together as giving some immediate relief; however, the relief did not last.

The participants also described therapists as sometimes conducting therapy with approaches that the participants found confusing, in the sense that they did not understand the presented theory, or why they were supposed to do the things the therapist prescribed. Some did not consider the therapeutic approach appropriate or helpful but seemed not to have considered the possibility of telling the therapist this. Some participants said that they thought the therapist knew best, despite their own growing sense of the futility of the approach. Some did not wish to offend, hurt, or disappoint their therapist, and even acted as if the therapy worked to please them. Yet, some just waited and hoped that it all would come to make sense to them or that the situation would resolve itself some way.

“We did an exercise today. Suddenly we were to go out of the room and just walk through the corridors and observe if anyone looked at us. I got annoyed at once because it was obviously going to fail with the two of us wandering about like baboons without any goal or purpose. I felt like that was completely unnecessary.”

All participants gave descriptions of their therapists in ways that often gave the impression of some distant person that had remained unknown to them and that had mostly not made them feel known, beyond being a patient. Somehow, the descriptions were either of the therapists or their own inner thoughts and feelings, rather than of the relationship itself, leaving a sense of two persons not having established an emotional bond.

#### Discovering the Possibility for Change and Development

In contrast to the above experiences, most participants also described, to a greater or lesser extent, experiences arising from treatment that exuded a sense of vitality, initiative, and movement. This sense of becoming an active participant in the treatment seemed connected to a sense of building trust and becoming understandable to themselves, through active efforts by the therapist to understand them. This activity and agency seemed to open up the possibility of development and change. Simultaneously, those participants who described this emerging development also expressed having to work hard to face their fears and insecurities, feeling that fear constituted the greatest barrier to change.

Participants who talked about vitality and movement in treatment described their therapists as having time and space for them. They considered that their therapists helped them to express themselves and as being interested and active. One participant said:

“She asked the right questions that I was able to answer.”

Furthermore, the therapists were described as willing to express their own thoughts and opinions, to give advice and guidance, show care and concern, as well as investment in the development of the participant. The participants’ descriptions thus reflected a sense of starting a process of sense making that seemed to move them toward both growing awareness and acceptance. This process seemed to imply a beginning of discovery of self and others.

“Sometimes my therapist says things that I have already thought about. Then I think that I am perhaps able to think a bit on my own? Sometimes she says; oh, I never thought about it like that, that is a nice way to think. If you have another person that can confirm or disconfirm that she has thought like that too, then I may trust my thoughts a bit more.”

Those participants who had taken part in various forms of group therapy described how they had been surprised to find that others would struggle with similar concerns, that others, who apparently fared well, could also suffer, and that there could be some common ground for all members. Even those participants who found it very challenging to take part and become visible in the group described this emergent discovery of others.

“It is horrible to be in the group. I just want to cry, my heart beats, I get a lump in my throat like I am going to throw up. It is like everybody is looking at me and thinking … but it is interesting to listen because they are there for a reason too. It is like a wake-up call that others might be like me.”

Just the observation of the dialogues between other group members seemingly made an impact. Some had to revise their initial skepticism, some had to adjust to surprisingly vulnerable reactions from others, and some found themselves missing the group, even if they had never managed to feel included.

“It helps to do something together. You build something together through sharing.”

The concrete content of the various therapeutic approaches of both individual and group modalities often seemed to take a less salient place in their experiences; however, most referred to the importance of learning new ways of thinking and perceiving themselves and others. Those who had taken part in more specialized psychotherapy would use the therapeutic nomenclature in a way that seemed to guide their sense making. These participants described how they had come to understand the connections between their developmental stories and their current functioning better and thus became more aware of their difficulties.

“In relation to maladaptive schemas… I have started to write up on all my high score schemas and started to think about what created them. Then I discuss with myself whether these schemata should hold any power over me today. Whether it was something that happened then and whether I can do something about it now so that they will not take control over me today.”

They explained how this awareness gave a sense of being able to manage themselves better, but that it also gave way to the need to find new practical solutions and strategies.

Together, these experiences of activity and movement were described as positive and associated with hope. Simultaneously, these participants conveyed a sense of standing on the edge of changing, trying to muster enough courage to try new ways of being. It was as if these engaged therapists would suggest possibilities at the same time as fear would pull the participants back. Being pulled back by this fear seemed connected to an experience of having to overcome on one’s own, like the awareness of connection with the therapist could become lost as their fear of novelty or change increased.

Discovery or new active learning hence came across as becoming possible within the context of interpersonal connection and a sense of trust, either in individual therapy or in a group setting. Simultaneously, connection and trust seemed to become more distant for the participants as their fear of change grew stronger, leaving them vulnerable to feeling lost and resorting to their familiar strategies of dealing with difficult thoughts and feelings, through disconnection and withdrawal. This oscillation between beginning trust and mistrust, as well as between connection and disconnection, seemed reflected in the tendency to both wish for prescribed solutions and being handled, as well as for the vitality that came with a beginning discovery of self, others, and agency. The participants thus searched for courage to do what frightened them the most, and the courage seemed within reach if they felt made understandable, accepted, and active.

## Discussion

The aim of this study was to inquire into how participants make sense of their experiences of treatment for AVPD. We present findings that convey an overarching theme of searching for courage to be. The participants expressed how their experience of being managed in treatment settings created, over time, a sense of discontentment and disconnection; however, they also told of experiencing the discovery of the possibility for change and development. Their goals were described as finding strength and trust within themselves, as well as freedom from the evaluations of others, thereby becoming able to choose what to feel, think, and do for themselves. To become able to do this, they perceived it necessary to overcome their fears and insecurities. Thus, their search for courage could be understood to involve finding a way to trust themselves to manage everyday life, through finding the strength not to let the judgment of others define their own state of mind, and to distinguish trustworthy, from not-so-trustworthy, others. This path could imply a courageous leap of faith in connecting to others, through believing that the other could accept and invite them into a sense of companionship while daring the risk of rejection. This courageous leap of faith emerged as essential for treatment, specifically for the therapeutic relationship in relation to opening up to acquiring new knowledge and attempting new behaviors. In the following section, we explore how these findings relate to theory, to further our understanding of the subjective experiences of treatment for AVPD.

Persons diagnosed with AVPD experience considerable ongoing fear and insecurity, and work hard to endure these, while longing for connection, both to themselves and others (Sørensen et al., 2019). The participants in this study expressed how they search for answers regarding how they can become able to overcome their fears and insecurities and accomplish their goals. The relational context of therapy did come across as being of utmost importance for the participants in this study and seemed related to their experience of their treatments. This aligns with the well-established moderate but reliable association between the quality of the relationship and outcomes in therapy (e.g., Ardito and Rabellino, 2011; Horvath, 2018; Noyce and Simpson, 2018). The interpersonal relation between the client and the therapist and the instrumental aspects of the therapy are considered to occur in a dynamic and complex interplay, evolving and changing over time (Norcross and Lambert, 2018).

Our findings do point toward challenges our participants faced in establishing a reciprocal therapeutic relationship in treatment. The main challenge seems related to entering a mutual complementary relationship in the first place. At the same time, as the quality of therapeutic relationship seemed crucial to the experiences of therapy, the participants came across as being rather unaware of how to approach this therapeutic challenge, which again could be seen as mirroring relational challenges in their everyday lives (withheld for anonymous review).

To take part in a reciprocal therapeutic relationship includes forming an alliance of agreement of tasks and goals, as well as an emotional bond (Bordin, 1979, 1994). This involves establishing interpersonal connection, communication, and collaboration that fosters both agreement and negotiation of rupture repairs that arise with empathic breaches and alliance fluctuations (Safran and Kraus, 2014; Horvath, 2018). Research on client experiences of treatment and the therapeutic relationship supports the importance of the therapist providing safety, containment, care, respect, equality, and insight, as well as promoting agency within a relationship that allows for both connection and communication of relational challenges (Levitt et al., 2016; Shepherd et al., 2016; Katsakou and Pistrang, 2018; Kverme et al., 2019).

Our participants rather described a sense of growing disconnection and detachment, as they seemed pending understanding from the therapist at the same time as struggling with making themselves understood. Rather than entering a collaborative exploration of their goals and the best way to approach these goals, they seemed to wait for the therapist to give answers and prescribe solutions and directions. As they received these solutions from the therapist, the sense of interpersonal disconnection seemingly grew. Their initial hope for help to achieve their goals of trust, strength, and freedom dwindled as prescription, rather than collaboration, about solutions characterized the relationship. Telling their therapists about these experiences came across as a non-option, as the participants seemingly complied. This experience of being managed could be understood within the context of becoming the receiver, or perhaps the object, of knowledge and understanding. You may experience being looked upon with a clinical gaze, which conveys a sense of being a case or an object of interest, perhaps something like a disorder, or as showing deviations from the norm, that may be cured or moved closer to the norm by another (Gardner, 2016). Perhaps related to a focus on “cure” or “symptom reduction,” the relationship becomes in danger of being that of observer and observed, thus being characterized by detachment and objectivity (Buber, 1937; Jaaskelainen, 2000). Thus, the findings may convey a therapeutic interpersonal pattern that initially reinforces an emphasis on symptom reduction without either being aware of how connection, understanding, and collaboration are lacking.

By contrast, when feeling understood and known as a person, the participants’ descriptions came across with a sense of emerging vitality, initiative, and movement. Thus, viewing the themes together points toward the importance of building emotional connection to foster a sense of trust and safety in the therapeutic relationship that allows for collaboration and risk taking when approaching the client’s goals (e.g., Spencer et al., 2019; Tsai et al., 2019). The participants that described positive qualities of the therapeutic relationship did experience their therapists as being warm and caring while giving time and space for them. These therapists were furthermore perceived as being active and genuine through giving guidance and conveying faith in the participant’s developmental potential. Intertwined in these descriptions were tales of possible new learning, related to ways of thinking and perceiving themselves and others; however, a lingering fear over possible consequences of rejection and failure remained.

Fear of rejection and feelings of inadequacy in interpersonal encounters are viewed as central to AVPD. It has been suggested that pseudo-alliance and compliance are phenomena that must be carefully considered by therapists forming alliances with clients who are interpersonally sensitive, avoidant, and shame prone but who are simultaneously longing for connection (Bender, 2005; Doran, 2016; Simonsen et al., 2019). In addition, when collaboration is overly emphasized there is the danger of fostering compliance, which can be mistaken for alliance, in particular in cases where the therapist and client set aside their own needs to attend to the other’s or strive to maintain harmony and avoid commenting on strains in the relationship (Doran, 2016). The findings of Strauss et al. (2006) in their study on early alliance, alliance ruptures, and symptom change for AVPD and obsessive-compulsive disorders exemplify this aspect. The authors emphasize the importance of alliance rupture repairs for better alliances and outcomes and vice versa when strains are unattended. They furthermore view their findings in light of the importance of establishing trust and collaboration early on in therapy. Our findings also support the importance of monitoring alliance ruptures and compliance during therapy with people diagnosed with AVPD, as our participants would not tell of their dissatisfaction with treatment. However, to enable clients diagnosed with AVPD to share implies a beginning sense of trust. Our findings appear to indicate that a sense of being understood and feeling known was associated with trust and what could be understood as an increased sense of acceptance, which opened up potential for collaboration.

Trust can be viewed as essential for new learning, change, and development in treatment (Langley and Klopper, 2005). Our participants mainly described an experience of being managed that was not initially considered negative, as they searched for help from someone believed to be competent and professional. They initially seemed willing to place their trust in the competence of the therapist. They may also have been aware of the rationality of information about new adaptive ways of thinking conveyed in therapy, while remaining unable to apply them, most likely reflecting that trust is a feeling state that includes affective, cognitive, and conative elements, influenced by past experiences, as well as our surrounding context (Baier, 1986). Past experiences of the participants may have influenced their expectations of being accepted, rejected, or harmed when revealing their vulnerability thus decreasing their willingness to give discretionary power to another (Baier, 2010).

When treated as an intentional being with agency, an attitude of possible trust came across in their descriptions, together with an openness to there being something relevant for them to learn. To develop and change in a way that leads to an experience of intentionality and agency can be described as becoming a subject in the eyes of another subject (Fonagy and Allison, 2014; Fonagy et al., 2015; Bateman et al., 2018). Inter-subjectivity can be understood as the space in which we become; the interdependency that makes room for the emerging self (Stolorow and Atwood, 1992; Atwood and Stolorow, 2014). It can be seen as a relationship of co-operation between subjects with personal engagement through recognition, interest, confirmation, and a sense of responsibility for the other (Buber, 1937; Jaaskelainen, 2000). Within this relationship, there is an idea that interactions can become something more than the sum of two individual perspectives; the interaction itself may become something autonomous; for example, in the co-creation of new meanings that might both influence and transform the participants (De Jaegher et al., 2017).

For this to occur, some therapists described by our participants seemingly made themselves experientially available to their clients. The same came across in the participants’ descriptions of their experiences of attending group therapy, where listening to others putting their experience into words opened up new ways of perceiving fellow group members. Perhaps the group setting provided a first opportunity for these participants to discover how sharing of experiences may lay the ground for a sense of belonging. Thus, not only does the therapist have to convey their experience of the client as a subject, but they must also convey themselves as subjects, to build a therapeutic relationship of possible trust; however, this does not necessarily mean self-disclosure, perhaps more an emphasis on the embodied subjective presence in the interpersonal engagement and attuning to the others’ goal-directed, intentional being (Gallagher and Zahavi, 2012). If therapists use observational language, that is, give voice to what they observe about their clients’ experiences, dreams, beliefs, motivations, and desires, as well as of their mutual relationship, they may add to a sense of discovering oneself as an intentional agent through this guidance in reflexive thinking about oneself and others (Banham and Schweitzer, 2017). The attuned observational language combined with non-verbal attunement matching with the client’s affective state and arousal could make the interaction less threatening (Havas et al., 2015). As we practice articulating our situated points of view and relate them to our actions and events of which we are part, we express both our agency and our sense of self (Angus, 2012). We talk together about our narratives of our experiences in a concrete and particular shared world of interactions and how we understand and respond to them (Gallagher and Zahavi, 2012). This implies that a therapist must emphasize and validate the experience of the other while conveying their own efforts to understand the meaning of that subjective reality (Stolorow and Atwood, 1992). A sense of courage to approach that which scares us the most (Rachman, 2004) may thus come about when your subjective reality is confirmed and articulated in a reciprocal therapeutic relationship.

### Limitations

The aim of this study was to further understand the subjective experiences of treatment of persons diagnosed with AVPD; hence, it did not investigate how therapy for AVPD works or the quality of outcomes. The findings are based on the participants’ descriptions of their subjective experiences and not on the feasibility of their actual treatments or of the qualities of their therapists. In addition, all participants were taking part in treatment at the time of the interviews. This could imply that our findings were influenced by these therapeutic contexts. Furthermore, the participants were recruited from an out-patient hospital setting and could hence be representative of a specific level of severity of personality functioning, descriptive of this specific treatment setting. Lastly, due to the inductive and ideographic nature of the study, the findings do not distinguish between the possible influence of participant characteristics, diagnostics, or traits, between various treatments, or between normality and pathology; however, these important topics could be the subject of future research studies.

## Conclusion

Although we cannot say that our findings are specific to AVPD, we may understand the participants’ subjective experience of treatment for AVPD as articulating: “Make me an agent in my own life, so that I can discover my intentions and myself; however, I meet you with great vulnerability, as this form of trust has, from earlier experiences, not generated trustworthy knowledge about me or the ways of the world.” Through creating the circumstances for trust to emerge in an attuned inter-subjective space, new experiences and new knowledge may be passed on from the therapist to the client. Perhaps giving way to the courage to open up to how fears and anxiety are always part of life but can be faced when met within a fellowship of acceptance and faith in our abilities to develop and learn. Further, through new experiences, new learning through successes and failures may bring a sense of growing strength, trust in oneself to manage, and the freedom that comes from knowing that, even if you do not manage everything, you are still acceptable.

## Data Availability Statement

The datasets generated for this study will not be made publicly available. The dataset is textual and consists of transcribed in-depth interviews that have been de-identified. The content is still personal, cannot be made anonymous without changing the content and if made publicly available participants would be able to recognize themselves. Furthermore, the transcripts are in Norwegian and only the quotes have been translated to English.

## Ethics Statement

The studies involving human participants were reviewed and approved by the Regional Ethics Committee, REK, Sør-Øst ref.nr. 2015/980. The patients/participants provided their written informed consent to participate in this study.

## Author Contributions

KS collected the data and took a leading role in data analysis and writing the manuscript. TW, EB, and MR contributed to developing the study design and the reflective and analytic phases of the project and to writing the article.

### Conflict of Interest

The authors declare that the research was conducted in the absence of any commercial or financial relationships that could be construed as a potential conflict of interest.
